# Effects of risk for bipolar disorder on brain function: A twin and family study

**DOI:** 10.1016/j.euroneuro.2017.03.001

**Published:** 2017-05

**Authors:** Genichi Sugihara, Fergus Kane, Marco M. Picchioni, Christopher A. Chaddock, Eugenia Kravariti, Sridevi Kalidindi, Fruhling Rijsdijk, Timothea Toulopoulou, Vivienne A. Curtis, Colm McDonald, Robin M. Murray, Philip McGuire

**Affiliations:** a Institute of Psychiatry, Psychology & Neuroscience, King׳s College London, De Crespigny Park, SE5 8AF, UK; b St Andrew׳s Academic Centre, King׳s College London, NN1 5BG, UK; c Department of Psychiatry, Clinical Science Institute, National University of Ireland, Galway, University Road, Galway, Ireland

**Keywords:** Bipolar disorder, Functional magnetic resonance imaging, Working memory, Twin, Genetic modeling

## Abstract

Bipolar disorder (BPD) is associated with altered regional brain function during the performance of cognitive tasks. The relative contribution of genetic and environmental risk factors for BPD to these changes has not yet been quantified. We sought to address this issue in a functional neuroimaging study of people who varied in their risk for BPD. Functional magnetic resonance imaging was used to study 124 subjects (29 twin and 9 sibling pairs with at least one member with BPD, and 24 healthy twin pairs) performing a working memory task. We assessed the influence of risk for BPD on regional brain function during the task in a two stage process. Firstly, we identified areas where there were group differences in activation. Secondly, we estimated the heritability and phenotypic correlation of activation and BPD using genetic modeling. BPD was associated with increased activation in the anterior cingulate, orbitofrontal, medial prefrontal, and left precentral cortices, and in the precuneus. Within these regions, activation in the orbitofrontal cortex rendered the most significant heritability estimate (*h*^2^=0.40), and was significantly correlated with BPD phenotype (*r*_*ph*_=0.29). A moderate proportion of the genetic influences (*r*_*g*_=0.69) acting on both BPD and on the degree of orbitofrontal activation were shared. These findings suggest that genetic factors that confer vulnerability to BPD alter brain function in BPD.

## 1 Introduction

Bipolar disorder (BPD) has a strong genetic etiological component (Muller-Oerlinghausen et al., 2002). Clinically, patients experience core mood pathology but also altered cognitive function, including impairments in working memory (Arts et al., 2008). Working memory deficits are also seen in patients’ unaffected relatives (Arts et al., 2008). Functional magnetic resonance imaging (fMRI) studies suggest that patients performing working memory tasks, show altered activation in several frontal and parietal cortical regions (Adler et al., 2004, Drapier et al., 2008, Monks et al., 2004, Townsend et al., 2010). Altered frontal activation is also seen in fMRI studies of working memory tasks in unaffected relatives, who are at familial (i.e., genetic and shared environmental) risk (Drapier et al., 2008, Thermenos et al., 2010), and in healthy people with a genetic risk allele for BPD (Bigos et al., 2010). Because working memory-related activation in healthy subjects is partially genetically influenced (Blokland et al., 2011), a key question emerges as whether altered working memory activation in bipolar patients and their relatives is linked to the genetic risk for the disorder, and could thus serve as an intermediate phenotype marker for BPD (Preston and Weinberger, 2005).

Twin studies are the best means of investigating the relationship between genetic and environmental risk and candidate intermediate phenotypes. They permit firstly the quantification of the relative influence of genetic and environmental factors on the candidate intermediate phenotype, and then on the phenotypic correlation between the intermediate phenotype and the disorder. This approach has identified significant relationships between the risk for BPD and alterations in white matter volume (van der Schot et al., 2009), frontal grey matter concentration (van der Schot et al., 2010), event-related potentials (Hall et al., 2009), and cognition such as working memory and IQ (Georgiades et al., 2016), with genetic factors the main source of these associations. Environmental risk for BPD has been linked to alterations in global grey matter volume (van der Schot et al., 2009) and peripheral inflammatory markers (Padmos et al., 2009).

The present study had two aims, firstly to investigate familial influences on differential regional activation in BPD during a working memory task. Secondly, to quantify the common genetic and environmental origins with BPD. On the basis of the existing literature, we hypothesized: (1) familial risk for BPD would be associated with altered activation in frontal and parietal regions; and (2) these alterations would be more related to genetic than to environmental factors. To test these hypotheses, we examined a sample of twin and sibling pairs with BPD, their unaffected co-twins and siblings, and healthy twin pairs. We used fMRI to assess signal change during a working memory task. In regions showing group differences in activation, genetic modeling was then used to quantify the impact of genetic and environmental sources of variation on activation, and the extent to which the co-variation between BPD risk and activation was due to genetic, common environmental, and unique environmental effects.

## 2 Experimental procedures

### 2.1 Participants and assessments

Probands with BPD and their relatives were recruited nationally from clinical services, patient support groups, national media and a study website. Healthy control twins were recruited from the Institute of Psychiatry, Psychology and Neuroscience Volunteer Twin Register and national media. One hundred and twenty-four individuals participated in the study; 7 monozygotic (MZ) twin pairs concordant for BPD, 14 MZ and 8 dizygotic (DZ) discordant twin pairs (BPD patients and their unaffected co-twins), 9 discordant sibling pairs (BPD patients and their unaffected siblings), and 18 MZ and 6 DZ healthy control twin pairs. In concordant twin pairs both members, and in discordant pairs only one member met DSM-IV criteria for BPD, while their co-twin or sibling was unaffected by BPD. Diagnoses were determined by a post-graduate qualified psychiatrist using a structured clinical interview, augmented with a systematic review of the medical records. Forty three patients had bipolar I, and two bipolar II. Twenty-eight patients with BPD had experienced psychotic episodes. Two patients from MZ concordant pairs and 1 from an MZ discordant pair had a previous history of alcohol dependence, and one patient met DSM-IV criteria for panic disorder. Healthy controls and unaffected twins and siblings were screened for mental disorders using the Schedules for Clinical Assessment in Neuropsychiatry (Wing et al., 1990). Control subjects who met criteria for an Axis I disorder at the time of assessment or had a personal or family history of BPD were excluded. Among unaffected co-twins, 4 met lifetime criteria for major depressive disorder, 2 for an anxiety disorder, and 1 for a history of alcohol dependence, but all were clinically well at assessment, and included. The probability that any discordant pair would subsequently become concordant for BPD was low, as an average of 17.4 (standard deviation [SD]=11.7) years in the MZ, and 19.8 (10.2) years in the DZ/sibling pairs had elapsed since the probands illness began. Exclusion criteria for all subjects included organic brain disease, significant head trauma, and drug or alcohol dependence in the 12 months before participation. Zygosity was confirmed by DNA analysis of blood or cheek swab samples. Full-scale IQ was assessed using Wechsler Adult Intelligence Scale-R (Wechsler, 1981) or Wechsler Abbreviated Scale of Intelligence (Wechsler, 1999), and standardized using the mean and SD of the control sample. Handedness and parental socio-economic status were assessed using the Annett Handedness Questionnaire (Annett, 1970) and the Standard Occupational Classification (Great Britain, 1995) respectively, and current mood using the Beck Depression Inventory (BDI) (Beck et al., 1961) and the Altman Self-Rated Mania Scale (ASRM) (Altman et al., 1997). All patients were clinically stable and had been taking regular medication for at least one month before participation. After ethics committee approval, written informed consent was obtained from all participants.

### 2.2 Demographic, clinical, and behavioral data analysis

Group effects were analyzed using a regression model with standard errors that are robust against familial correlations (Binder, 1983, Picchioni et al., 2010). Regression and logistic regression with the Huber–White sandwich standard error were used to compare demographic, clinical, and behavioral variables while taking account of family clusters. We carried out overall group comparisons followed by *post-hoc* pair-wise comparisons where the initial test was significant using established testing models (Picchioni et al., 2006, Picchioni et al., 2010) in STATA 10.1.

### 2.3 MRI data acquisition

The subjects were scanned performing a modified sequential letter N-back working memory paradigm (Barch et al., 1997). The task involved blocks of four conditions: three active (1-, 2-, and 3-back) and baseline (0-back), three of each active condition and nine 0-back baseline blocks, giving 18 in total. Each 30-sec block consisted of 15 presentations of a single letter stimulus. Response accuracy and reaction time were recorded in real time. Gradient-echo echo-planar images were acquired at 1.5 T on a single GE Signa system. Two hundred and seventy T2*-weighted images depicting the blood-oxygenation-level-dependent (BOLD) contrast were acquired: echo time 40 msec; repetition time 2 sec; in-plane resolution 3.44 mm; slice thickness 7 mm; interslice gap 0.7 mm; flip angle 70°; matrix 64×64; field of view 24 cm.

### 2.4 Image analyses

Preprocessing and individual level analysis were performed in Statistical Parametric Mapping software (SPM5; http://www.fil.ion.ucl.ac.uk/spm/). All volumes from each participant were realigned to the first reference volume, resampled to a voxel size of 2×2×2 mm^3^, normalized to a standard template, and then spatially smoothed (8-mm full width at half maximum isotropic Gaussian kernel). Subject specific models were generated for each participant by convolving each onset time with a synthetic hemodynamic response function. Baseline and the 3 active conditions were modeled separately. The general linear model implemented in SPM was used to calculate parameter estimates across all voxels. In this study, we present only the 2-back versus 0-back contrast, as previous work has indicated that this represents the optimal task load to investigate working memory-related brain activity (Callicott et al., 1999) and the genetic liability for the disorder with this task (Drapier et al., 2008).

To address within-family correlations within the fMRI data, for the MZ BPD concordant, MZ and DZ control pairs, that is, the groups where both members of each twin pair share the same clinical phenotype, a single pair level map was created by averaging functional parameter estimates between the two members of the pair. Then, this pair level map was entered into the second level analysis rather than each subject individually. We used a permutation-based non-parametric method to establish the effect of experimental group on regional brain activity across the entire brain (http://www.kcl.ac.uk/ioppn/depts/neuroimaging/research/imaginganalysis/Software/XBAM.aspx). Subjects were divided into 6 experimental groups based on zygosity, clinical status, and the relationship of the subject to the proband (Table 1). Using the Jonckheere-Terpstra test, we assessed if there was a monotonic (but not necessarily linear) trend in the BOLD parameter estimates across the 6 groups. Response accuracy and IQ were entered as covariates for all between-group testing. Hypothesis testing was carried out at the cluster level using permutation testing against the null hypothesis (Bullmore et al., 1999). In order to reduce the multiple comparison problem, hypothesis testing was carried out at the cluster level (Bullmore et al., 1999), using voxels that exceeded a probability threshold (*p*<0.01 uncorrected) were retained for analyses. All analyses are reported at an adapted cluster-level threshold set to less than one false positive cluster.

**Table 1:** Demographics and clinical characteristics and task performance.

	MZ Cc ill	MZ Dc ill	DZ/Sibl Dc ill	MZ Dc unaffected	DZ/Sibl Dc unaffected	All control twins	Groupcomparison *F* or *χ*^2^ (df) *p*
No of participants	14	14	17	14	17	48	
Age, mean (SD)	38.4 (11.6)	40.4 (14.5)	43.5 (11.6)	40.6 (14.5)	42.8 (11.6)	35.6 (11.4)	1.31 (5,61)
							0.271
Female/male	4/10	11/3	9/8	11/3	8/9	38/10	9.09 (4)
							0.059
Education,years, mean (SD)	13.5 (2.4)	15.6 (2.5)	15.5 (2.5)	16.2 (2.9)	16.2 (3.5)	15.6 (2.5)	1.46 (5,61)
							0.215
Parental social class, mean (SD)^a^	3.7 (1.0)	2.6 (1.2)	2.4 (1.3)	2.4 (0.9)	2.2 (0.9)	2.6 (1.3)	3.46 (5,61)
							0.008
Handedness,right/left/mixed	12/1/1	11/2/1	16/1/0	13/1/0	16/0/1	43/2/3	2.63 (5)
							0.756
IQ^a^	98.7 (7.6)	113.9 (10.1)	119.2 (10.1)	115.1 (8.7)	120.6 (7.0)	115.3 (11.4)	10.08 (5,61)
							<0.001
BDI^b^	9.6 (8.9)	14.0 (10.8)	8.4 (5.9)	4.6 (4.2)	2.2 (3.6)	3.3 (2.9)	8.40 (5,61)
							<0.001
ASRM^b^	3.5 (3.5)	3.9 (2.9)	3.8 (2.9)	2.6 (2.7)	1.4 (1.8)	2.9 (2.3)	2.57 (5,61)
							0.036
2-back Accuracy^c^	81.3 (28.9)	71.4 (24.7)	66.9 (38.3)	87.5 (13.0)	93.4 (12.6)	89.3 (15.3)	2.92 (5,61)
							0.020
2-back Reaction time	0.737 (0.126)	0.812 (0.214)	0.579 (0.300)	0.711 (0.150)	0.864 (0.150)	0.684 (0.183)	1.61 (5,61)
							0.170

Abbreviations: MZ, monozygotic; DZ, dizygotic; Sibl, siblings, Cc, concordant; Dc, discordant; BDI, the Beck Depression Inventory; ASRM, the Altman Self-Rated Mania Scale; Li, Lithium; VPA, valproic acid; CBZ, carbamazepine.

a MZ concordant ill group had a significantly higher social class and a significantly lower IQ compared to other groups (*p*<0.05).

b Patient group had higher scores in BDI and ASRM than non-patient group (*p*=0.001 and 0.044 for the BDI and ASRM, respectively).

c Patient group had a significantly lower accuracy compared to unaffected siblings and controls.

From the clusters identified in the second level between-group analysis, we created a region of interest mask, then extracted the mean BOLD parameter estimates (for the 2-back>0-back contrast) for each cluster from each individual׳s normalized data, which was used as the index of regional brain activation in the subsequent genetic modeling analysis.

### 2.5 Genetic modeling

Genetic modeling was used to investigate the relative genetic, common and unique environmental effects on the extracted regional brain activation from the clusters identified in the second level analysis, namely: the anterior cingulate cortex, orbitofrontal cortex, medial prefrontal cortex, precuneus, and the left precentral gyrus (see Section 3).

We fitted a correlational model to estimate the MZ and DZ/sibling correlations for BPD and regional brain activation after fixing the win correlations for BPD (*r*_*MZ*_=0.85 and *r*_*DZ*_=0.425) (McGuffin et al., 2003) and the population lifetime prevalence (1%) (Bebbington and Ramana, 1995). The correlational model then yielded (1) cross-member within-trait (for example, BPD status in subject1 with BPD status of their twin or sibling) and (2) cross-member cross-trait (for example, BPD status in subject1 with brain activation of their twin or sibling) correlations.

The genetic models for BPD and regional brain activation then separate the variance of each measure into their respective genetic and environmental components. Additive genetic factors (heritability, *h*^2^) represent the effects of genes; common environment (*c*^2^) represents shared environmental factors within the family, while unique environment (*e*^2^) represents environmental factors that make members of the same family different, e.g., accidents. The models estimate *h*^2^, *c*^2^, and *e*^2^ based on the cross-member cross-trait correlations.

Finally, partitioning any covariation between BPD and brain activation into its genetic and environmental sources yielded a genetic (*r*_*g*_) and unique environmental (*r*_*e*_) correlation. *r*_*g*_ is the extent to which the same genetic effects influence BPD and brain activation, while *r*_*e*_ is the degree to which the same unique environmental effects are common to both. Combining the data for *r*_*g*_ and *r*_*e*_ with *h*^2^ and *e*^2^ in the last step of the analysis allowed us to partition any correlations between BPD and brain activation (*r*_*ph*_) into its genetic (*r*_*ph-a*_) and unique environmental (*r*_*ph-e*_) components.

Prior to genetic modeling analyses, the effects of age and gender were regressed out. BPD status and the extracted parameter estimates were modeled as threshold traits. We performed structural equation modeling with maximum likelihood estimation of parameters using Mx (http://www.vcu.edu/mx/). A goodness-of-fit index (χ^2^) was obtained by computing the difference in likelihoods and the degrees of freedom between the genetic model and a model from which the genetic and common environmental components were dropped.

## 3 Results

### 3.1 Demographics, clinical characteristics, and task performance

The participants’ demographic and clinical characteristics, and task performance are summarized in Table 1 and Supplementary Table S1. There were significant group differences (*p*<0.05) in parental social class and IQ, driven by relatively low values in the MZ twins discordant for BPD. As expected, patients had higher BDI and ASRM scores, and made more errors than the unaffected groups on the 2-back condition (data not shown).

### 3.2 Neuroimaging

#### 3.2.1 Task-related activation in each group

**Figure 1:**
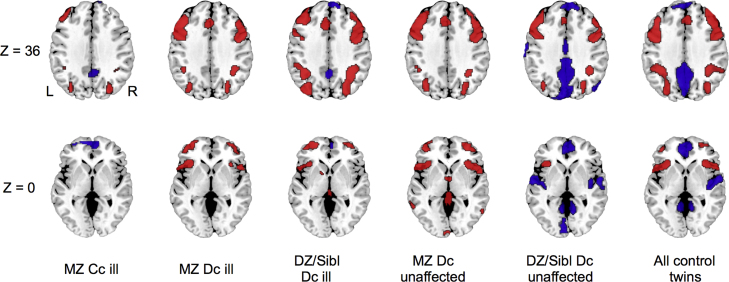
Regional brain activation (red) and deactivation (blue) in each group during the 2-back task compared to 0-back condition. Voxel-wise probability was thresholded at *P*<0.01 and cluster-wise probability was thresholded at <1 false positives. *Z*-coordinates refer to the axial level in MNI space. All groups displayed activation in the lateral prefrontal and posterior parietal cortex. Abbreviations: MZ, monozygotic; DZ, dizygotic; Sibl, siblings; Cc, concordant; Dc, discordant; L, left; R, right.

A qualitatively similar pattern of activation involving the lateral prefrontal and posterior parietal cortices was evident in all groups (Figure 1), although these varied in the topographical extent of activation. Four of the 6 groups showed deactivation in medial prefrontal and posterior cingulate cortex, while the unaffected DZ/siblings and control twins groups also showed deactivation in the middle temporal gyrus. These results did not show any activation or deactivation which are incompatible with previous studies, implying that the thresholds used in the neuroimaging analyses may not be liberal. Thus, we applied these thresholds to the imaging analyses throughout.

#### 3.2.2 Between-group testing – all groups

**Figure 2:**
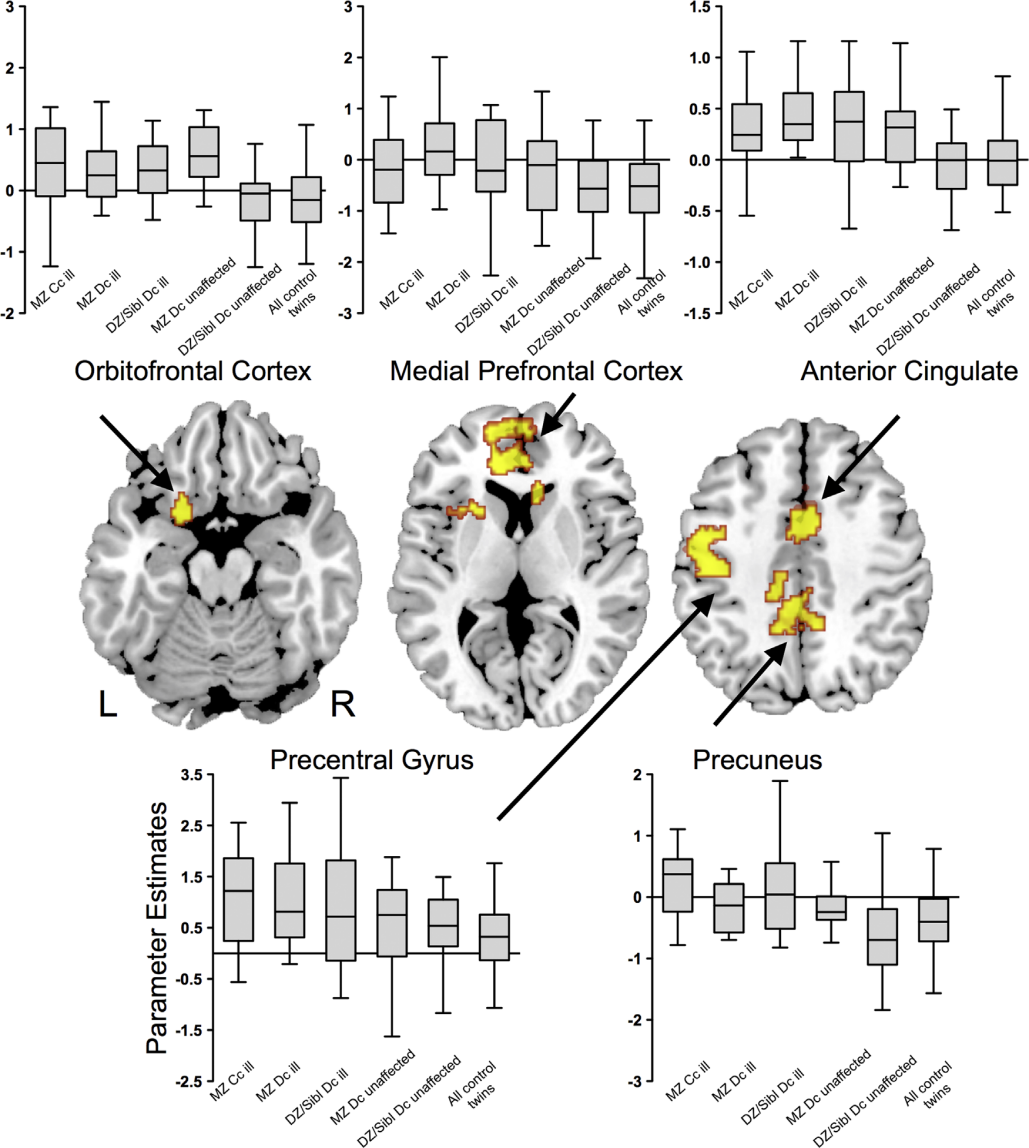
Effects of bipolar status on activation during the 2-back task across all 6 groups. There were significant differences in activation in the orbitofrontal, medial prefrontal, anterior cingulate, precentral and medial parietal cortex. Box-plots indicate extracted parameter estimates in each region. Voxel-wise probability was thresholded at *p*<0.01 and cluster-wise probability to deliver <1 false positive per contrast. Abbreviations: L, left; R, right.

There were 5 clusters where activation differed significantly across the 6 groups (Figure 2, Table 2). These comprised (1) the dorsal anterior cingulate/paracingulate cortex bilaterally, (2) the left orbitofrontal cortex/striatum/thalamus/insula, (3) the medial prefrontal/ventral anterior cingulate cortex bilaterally, (4) the precuneus/posterior cingulate cortex bilaterally, and (5) the left pre/postcentral gyrus.

**Table 2:** Monotonic analysis in 2-back vs. 0-back task across all participants and non-patient groups.⁎

**No of voxels**	**Regions**	**MNI coordinates of the centroid voxel**		
		***x***	***y***	***z***
**All participants**				
742	Bilateral Dorsal Anterior Cingulate Cortex/Bilateral Paracingulate Cortex	10	28	12
726	Left Orbitofrontal Cortex/Left Striatum/Left Thalamus/Left Insula	−20	10	−16
797	Bilateral Medial Prefrontal Cortex/Bilateral Ventral Anterior Cingulate Cortex	−12	60	8
872	Bilateral Precuneus/Left Posterior Cingulate Cortex	−6	−46	58
728	Left Precentral Gyrus/Left Postcentral Gyrus	−50	−4	50

**Non-patient groups**				
667	Bilateral Thalamus/Left Orbitofrontal Cortex/Right Amygdala/Left Caudate	−2	4	−4
331	Bilateral Dorsal Anterior Cingulate Cortex	6	20	18
508	Left Posterior/Anterior Cingulate Cortex	−16	4	32
446	Left Middle/Superior Temporal Gyri	−56	−12	−14

Abbreviations: MNI, Montreal Neurological Institute.

⁎ Cluster-wise probability was thresholded at <1 false positive.

#### 3.2.3 Between-group testing: non-patient groups

**Figure 3:**
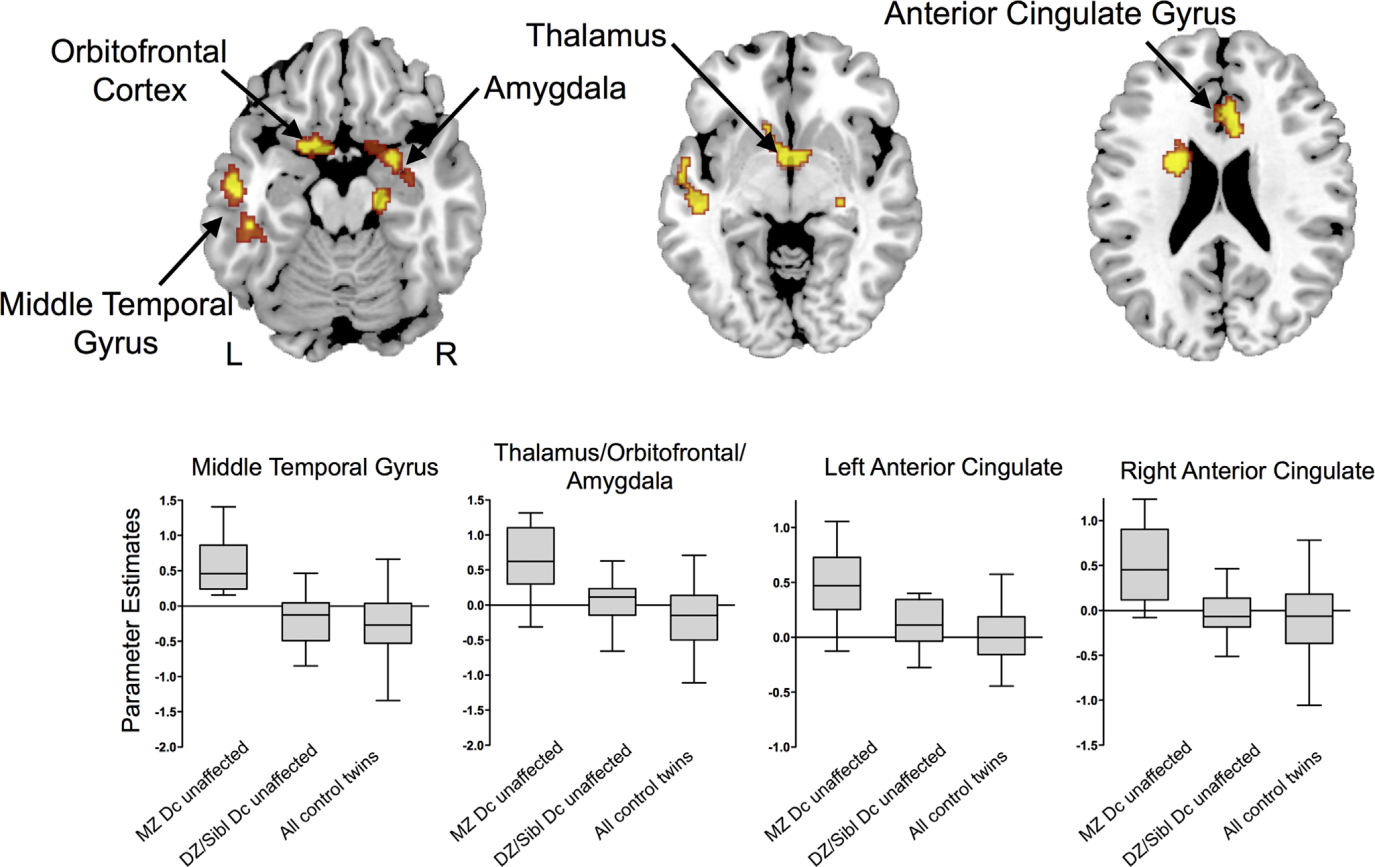
Effects of bipolar status on activation during the 2-back task in the three groups that did not include patients with BPD (MZ discordant unaffected, DZ & Siblings discordant unaffected, and all control twins). The groups showed significant differences in activation in the temporal, orbitofrontal, and anterior cingulate cortex. Box-plots indicate extracted parameter estimates in each region. Voxel-wise probability was thresholded at *p*<0.01 and cluster-wise probability at <1 false positive. Abbreviations: L, left; R, right.

When the analysis was restricted to the three non-patient groups, the MZ discordant unaffected, DZ & Siblings discordant unaffected, and MZ and DZ control twins, there were significant differences in activation in the dorsal anterior and posterior cingulate, left orbitofrontal and middle temporal cortices, the right amygdala, and thalami (Figure 3, Table 2). The anterior and posterior cingulate and orbitofrontal differences were located in similar regions to the differences that were found across all 6 groups (Figure 2).

#### 3.2.4 Effects of potential confounding factors

There were no significant effects of gender, parental social class, BDI or ASRM score on the extracted parameter estimates from any cluster. Although in patients there was an overall effect of psychotropic medication on activation in the left precentral gyrus (*p*<0.001), there were no significant differences in the parameter estimates between patients who were and were not receiving either mood stabilizers, antipsychotics, or antidepressants for any cluster. No significant differences in task performance or in the extracted parameters were found between patients with and without psychotic episodes. Repeating the analyses after excluding patients with (1) bipolar II, (2) a history of alcohol dependence, (3) comorbid panic disorder, or (4) non-BPD subjects with history of any psychiatric disorder did not significantly alter the results.

### 3.3 Genetic modeling

**Figure 4:**
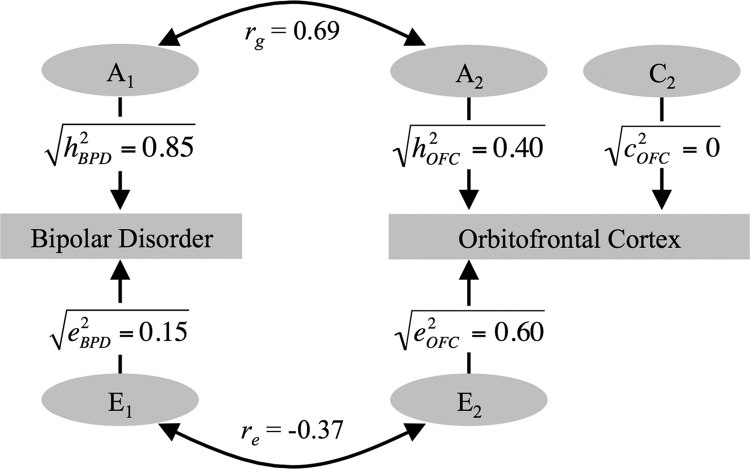
The correlated-factors solution of the genetic model for the orbitofrontal (OFC) cluster. The additive genetic (A_1_ and A_2_) and unique environmental (E_1_ and E_2_) factors on bipolar disorder (BPD) and brain activation in the OFC are correlated (*r*_*g*_, *r*_*e*_). The paths from A_1_ to bipolar disorder and A_2_ to brain activation are the square roots of their heritabilities (*h*^2^). Part of the phenotypic correlation (*r*_*ph*_) due to genetic effects is calculated by √0.85×*r*_*g*_×√h^2^_OFC_ and that due to environmental effects by √0.15×*r*_*e*_×√*e*^2^_OFC_. The C_1_ factor was omitted because *c*^2^ for bipolar disorder was fixed to zero.

The maximum likelihood correlations are given in Table 3. We restricted the subsequent genetic modeling to the orbitofrontal cortex, because it was the only region where the MZ correlation was significant. Additive genetic effects (*h*^2^=0.40) and unique environmental influences (*e*^2^) were significant. There was a significant positive phenotypic correlation (*r*_*ph*_=0.29) between increased activation in the orbitofrontal cortex and increased risk for BPD. Furthermore, brain activation in this cluster showed a significant genetic correlation with BPD (*r*_*g*_=0.69), with genetic factors providing a significant source of the correlation between the two (*r*_*ph-a*_=0.4) (Table 4). The solution of the genetic model for brain activation during working memory in the orbitofrontal cluster is illustrated in Figure 4. The model gave a good fit (∆*χ*^2^(df=2)=16.9, *p*<0.001).

**Table 3:** Cross-member within-trait and cross-member cross-trait correlations (*r* and 95% CI).

	Correlation of brain activation within members of twin and sibling pairs		Correlation of brain activation with bipolar disorder across members of twin and sibling pairs	
	MZ	DZ and siblings combined	MZ	DZ and siblings combined
Anterior Cingulate	0.25	0.39	0.30	0.01
	(−0.09 to 0.54)	(−0.08 to 0.72)	(0.11 to 0.47)	(−0.23 to 0.25)
Precentral Gyrus	0.24	−0.14	0.28	0.02
	(−0.12 to 0.54)	(−0.55 to 0.31)	(0.08 to 0.46)	(−0.24 to 0.29)
Orbitofrontal Cortex	0.48	−0.30	0.37	0.01
	(0.17 to 0.71)	(−0.65 to 0.16)	(0.18 to 0.53)	(−0.23 to 0.27)
Medial Prefrontal Cortex	0.22	0.10	0.17	−0.05
	(−0.13 to 0.53)	(−0.37 to 0.52)	(−0.02 to 0.34)	(−0.29 to 0.20)
Precuneus	0.26	0.02	0.36	−0.04
	(−0.09 to 0.55)	(−0.41 to 0.45)	(0.17 to 0.52)	(−0.27 to 0.22)

Abbreviations: CI, confidence interval; MZ, monozygotic; DZ, dizygotic. CIs including zero indicate non-significance.

**Table 4:** Parameter estimates, CIs and goodness-of-fit from the genetic modeling for the cluster that included the orbitofrontal cortex.

Variance components			Covariance components			Geneticandenvironmental correlations		Goodness-of-fit index^a^
*h*^2^	*c*^2^	*e*^2^	*r*_*ph*_	*r*_*ph-a*_	*r*_*ph-e*_	*r*_*g*_	*r*_*e*_	∆*χ*^2^ (*P*)
0.40	0.00	0.60	0.29	0.40	−0.11	0.69	−0.37	16.9 (<0.001)
(0.09–0.65)	(0.00–0.30)	(0.35–0.89)	(0.13–0.44)			(0.35–1.00)	(−0.68 to 0.02)	

Parameters for bipolar disorder are fixed based on a prevalence of 1% and the following genetic model: *h*^2^=0.85, *c*^2^=0, *e*^2^=0.15. Abbreviations: CI, confidence interval; *h*^2^, additive genetic effects; *c*^2^, shared environment effects; *e*^2^, unique environment effects; *r*_*ph*_, total phenotypic correlation; *r*_*ph-a*_, breakdown of the phenotypic correlation into genetic components; *r*_*ph-e*_, breakdown of the phenotypic correlation into environmental components; *r*_*g*_, genetic correlation; *r*_*e*_, unique environmental correlation. CIs including zero indicate non-significance.

a A ∆*χ*^2^ with a significant *p* value indicates a good fit.

As the orbitofrontal cluster was large and extended into adjacent regions, we repeated the analyses after resolving the cluster into component subclusters (Rubia et al., 2009) to test if the findings were specific to the orbitofrontal cortex *per se*. After resolution, the orbitofrontal subcluster remained both the largest and most significant cluster (Supplementary Figure S1 and Table S2). Using these sub-clusters as a mask, we repeated the parameter estimate extraction and the genetic modeling. There was still a significant correlation of activation within MZ pairs in the orbitofrontal subcluster, but not in any of the other 8 subclusters (Supplementary Table S3). Within the orbitofrontal subcluster, there was a significant genetic influence on activation, and this genetic influence was significantly correlated with genetic risk for BPD (Supplementary Table S4).

## 4 Discussion

We investigated the association between alterations in regional brain function and bipolar disorder and its familial risk, using a working memory paradigm. We were able to quantify the relative contributions of genetic and environmental influences to these functional changes. Consistent with our hypothesis, BPD status was associated with impaired working memory performance and increased activation in frontal, cingulate, and parietal cortical regions. Genetic modeling analyses showed that there was a significant correlation between BPD status and increased activation in the orbitofrontal cortex during working memory engagement, attributable in part to shared genetic effects. These findings suggest that the alteration in function in this region during working memory processing is related to the genetic liability for BPD. Put another way, some of the genes responsible for BPD also influence increased brain activation in the orbitofrontal cortex during working memory performance.

The effect of BPD status was evident in clusters that included the anterior cingulate cortex, orbitofrontal cortex, medial prefrontal cortex, precuneus, and precentral gyrus. The anterior cingulate cortex and precentral gyrus are robustly activated when healthy subjects perform the N-back task (Owen et al., 2005), with links to attentional and effort-related processing (Callicott et al., 1999), and the maintenance of visuospatial attention (Owen et al., 2005), respectively. However, we also found evidence that the findings in the precentral gyrus may be related to effects of medication.

The orbitofrontal cortex is implicated in emotional processing and regulation (Bechara et al., 2000, Davis and Whalen, 2001) and may play an emotional gating role in the context of working memory tasks (Pochon et al., 2002). Our findings in the orbitofrontal cortex are in line with previous studies in BPD which found an association between the risk for BPD and alterations in brain function (Thermenos et al., 2010) and structure (van der Schot et al., 2010) in this region. The cluster of activation that included the orbitofrontal cortex extended into adjacent structures that included the thalamus, striatum, and insula, regions that are also implicated in the pathophysiology of BPD (Cannon et al., 2007). The medial prefrontal cortex and precuneus are conceptualized as components of default mode network (Raichle et al., 2001). Regional activation in this network is thought to be influenced by genetic factors (Glahn et al., 2010), and is disrupted in BPD (Costafreda et al., 2011, Ongur et al., 2010).

In each of these regions, there was a positive correlation between brain activation and BPD status, with familial risk being associated with an intermediate increase in activation, and having BPD associated with an even greater degree of activation. This suggests that progressively greater activation may have been required to maintain task performance as the level of risk increased from DZ twins and siblings, through MZ twins to the patients. A similar pattern of increased activation has been found in studies of other cognitive (Allin et al., 2010) and emotional (Surguladze et al., 2010) tasks in unaffected relatives of BPD patients, and is consistent with a cortical ‘inefficiency’ model during working memory in people at risk of and with BPD (Bigos et al., 2010).

By restricting the analysis to the three non-patient subgroups, we were also able to examine the effect of familial risk in the absence of the potentially confounding effects of symptoms and treatment. The results resembled those that included the patients, with increasing risk for BPD being associated with greater activation in a similar set of areas. However, in the non-patient subsample there were additional effects in the amygdala and middle temporal gyrus that were not evident in the larger sample. This difference may be explained by environmental effects unique to the patient groups such as medication.

Although the dorsolateral prefrontal cortex (DLPFC) is implicated in working memory tasks (Owen et al., 2005) and has been linked to the pathophysiology of BPD, we found no effects of BPD status on activation in that region. However, an absence of DLPFC activation differences during working memory tasks has been reported in previous studies of the unaffected relatives of patients with BPD (Drapier et al., 2008, Thermenos et al., 2010).

We sought to quantify the strength of the relationships between BPD and regional activation. In the orbitofrontal cluster, the cross-member within-trait and cross-member cross-traits correlations for MZ twins were both significant and greater than that for the DZ twin/sibling group, suggesting that common familial etiological influences linked BPD and the strength of regional activation in that region. The heritability estimate in the orbitofrontal cortex was significant (*h*^2^=0.40), while a moderate (*r*_*g*_=0.69) proportion of the genetic influences acting on BPD and orbitofrontal activation overlapped. Taking the heritability (*h*^2^) into account, the phenotypic correlation (*r*_*ph*_) between BPD and orbitofrontal activation was in large part attributable to shared genetic influences (*r*_*ph-a*_). These findings strongly suggest that the genetic factors that drive a proportion of the changes in activation in that region are in fact the same as the genes responsible for the genetic liability to the disorder. Further analyses confirmed that the findings in the large orbitofrontal cluster were indeed localized to the orbitofrontal cortex *per se* (Supplementary Figure S1).

One limitation of the present study is that within-family correlations violate the independence assumptions of existing image analysis methods. In order to minimize the effects of within-family correlations, for each twin pair where both members shared the same clinical status and thus belonged to the same experimental group, we created a single mean twin map that served as the input to the second level analysis. Although variation in mood and the presence of psychotropic medications can influence brain activation (Bell et al., 2005, Blumberg et al., 2003), our analyses indicated that medication effects were limited to the precentral cortex, and that activation was not correlated with level of mood. The relatively wide confidence intervals in the genetic modeling (indicating uncertainty in the point estimates of genetic and environmental influences) suggest that we may not have had sufficient statistical power to detect correlations in all brain areas. However, to our knowledge, the present study is the largest of its kind (i.e., a functional neuroimaging study) to date.

In conclusion, the results from this study confirm that risk for BPD is associated with alterations in frontal, cingulate and parietal function. Our findings also suggest that variance in brain activation during working memory in the orbitofrontal cortex is primarily driven by the genetic risk for BPD.

## Role of funding source

Mitsubishi Pharma Research Foundation, Japan, and Research Training Fellowship from the Wellcome Trust had no further role in study design; in the collection, analysis and interpretation of data; in the writing of the report; and in the decision to submit the paper for publication.

## Contributors

GS, FK, MMP, and PM designed the study. FK, CAC, EK, SK, VAC, and CM acquired the data, which GS, FK, MMP, FR, and TT analyzed. GS, FK, MMP, RMM, and PM wrote the article, which all authors reviewed and approved for publication.

## Conflict of interest

The authors declare that they have no conflict of interest.
