# Supplementary material for: Effects of risk for bipolar disorder on brain function: A twin and family study
Source: Eur Neuropsychopharmacol. 2017 May;27(5):494–503. doi: 10.1016/j.euroneuro.2017.03.001 (PMC5446324; doi:10.1016/j.euroneuro.2017.03.001)
Supplement: Supplementary file 1 — Supplementary material [file mmc1.doc]

**Supplementary Figure S1**

**
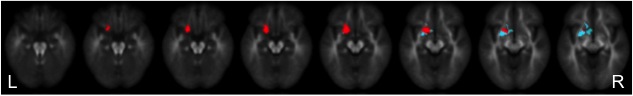
**

The orbitofrontal subcluster (red). Light blue indicates adjacent regions in the original orbitofrontal cluster.

|  | MZ Cc ill | MZ Dc ill | DZ/Sibl Dc ill |
| --- | --- | --- | --- |
| N of participants | 14 | 14 | 17 |
| N of patients with the psychotic features | 11 | 11 | 6 |
| Lithium use, No.  mean dose (range) | 6  783  (800-1200) | 2  900  (800-1000) | 9  822  (400-1000) |
| VPA use, No.  mean dose (range) | 4  1150  (600-2000) | 1  1000 | 3  1300  (1250-1400) |
| CBZ use, No.  mean dose (range) | 1  600 | 5  420  (200-800) | 2  750  (500-1000) |
| Antipsychotic use, No. (atypical/typical)  mean dose (range)^a^ | 10 (9/1)  340  (100-600) | 3 (2/1)  180  (50-390) | 4 (3/1)  92  (25-200) |
| Antidepressant use, No.  mean dose (range)^b^ | 0 | 10  28  (5-60) | 1  20 |
| Lamotrigine use, No.  mean dose (range) | 0 | 0 | 2  150  (100-200) |
| Medication free, No. | 4 | 2 | 4 |

**Table S1. Clinical Characteristics of Patients**

^a^ chlorpromazine equivalent. ^b^ fluoxetine equivalent.

**Supplementary Table S2**

Peak Coordinates, Cluster Size, and P-value in the Original Orbitofrontal Cluster

| Region | L OFC | L Thalamus | L Putamen | L Caudate | L Putamen | L Caudate | L Putamen | L Putamen | L Insula |
| --- | --- | --- | --- | --- | --- | --- | --- | --- | --- |
| MNI coordinates  (x, y, z) | -20, 10, -16 | -6, 0, -2 | -22, 14, 0 | -6, 14, -2 | -24, 16, -8 | -10, 20, -4 | -24, 2, -8 | -24, 2, -6 | -32, 14, 8 |
| Cluster size (voxel) | 184 | 136 | 21 | 137 | 29 | 111 | 66 | 28 | 15 |
| *p* value of Maximum | 0.000006 | 0.000184 | 0.001302 | 0.000139 | 0.000861 | 0.000094 | 0.000184 | 0.000184 | 0.00133 |

The original orbitofrontal cluster was de-clustered using a function of in house software. Abbreviations: L, left; OFC, orbitofrontal cortex

**Supplementary Table S3**

**Cross-member Within-trait and Cross-member Cross-trait Correlations (*r* and 95% CI) of the Orbitofrontal Subcluster**

|  | Correlation of brain activation within members of twin and sibling pairs | | Correlation of brain activation with bipolar disorder across members of twin and sibling pairs | |
| --- | --- | --- | --- | --- |
| Region  (coordinates) | MZ | DZ and siblings combined | MZ | DZ and siblings combined |
| L OFC  (-20, 10, -16) | 0.36  (0.02 – 0.63) | -0.12  (-0.55 – 0.35) | 0.35  (0.17 – 0.52) | 0.002  (-0.25 – 0.26) |
| L Thalamus  (-6, 0, -2) | 0.20  (-0.16 – 0.51) | -0.48  (-0.76 – -0.05) | 0.07  (-0.11 – 0.25) | 0.12  (-0.11 – 0.35) |
| L Putamen  (-22, 14, 0) | 0.17  (-0.16 – 0.48) | -0.09  (-0.51 – 0.37) | 0.17  (-0.16 – 0.48) | -0.08  (-0.31 – 0.17) |
| L Caudate  (-6, 14, -2) | 0.29  (-0.06 – 0.58) | -0.39  (-0.72 – 0.06) | 0.20  (0.02 – 0.37) | 0.14  (-0.10 – 0.38) |
| L Putamen  (-24, 16, -8) | 0.24  (-0.09 – 0.30) | -0.23  (-0.61 – 0.23) | 0.27  (0.09 – 0.44) | 0.04  (-0.21 – 0.30) |
| L Caudate  (-10, 20, -4) | 0.13  (-0.21 – 0.45) | 0.09  (-0.37 – 0.51) | 0.27  (0.09 – 0.43) | 0.05  (-0.20 – 0.29) |
| L Putamen  (-24, 2, -8) | 0.28  (-0.06 – 0.56) | 0.14  (-0.32 – 0.54) | 0.29  (0.10 – 0.46) | 0.19  (-0.07 – 0.42) |
| L Putamen  (-24, 2, -6) | 0.23  (-0.13 – 0.54) | 0.19  (-0.28 – 0.58) | 0.03  (-0.16 – 0.23) | -0.01  (-0.25 – 0.24) |
| L Insula  (-32, 14, 8) | 0.26  (-0.09 – 0.56) | 0.09  (-0.35 – 0.50) | 0.30  (0.11 – 0.47) | 0.03  (-0.21 – 0.27) |

Abbreviations: CI, confidence interval; MZ, monozygotic; DZ, dizygotic; OFC, orbitofrontal cortex. CIs including zero indicate non-significance.

**Supplementary Table S4**

**Parameter Estimates, CIs and Goodness-of-Fit from the Genetic Modeling for the Orbitofrontal Subcluster**

| Variance components | | | Covariance components | | | Genetic and environmental correlations | | Goodness-of-fit index ^a^ |
| --- | --- | --- | --- | --- | --- | --- | --- | --- |
| h^2^ | c^2^ | e^2^ | *r_ph_* | *r_ph-a_* | *r_ph-e_* | *r_g_* | *r_e_* | ∆χ^2^  (*p*) |
| 0.33  (0.06 – 0.60) | 0.00  (0.00 – 0.35) | 0.67  (0.40 – 0.92) | 0.22  (0.06 – 0.38) | 0.37 | -0.15 | 0.70  (0.32 – 1.00) | -0.47  (-0.76 – -0.09) | 14.7  (0.002) |

Parameters for bipolar disorder are fixed based on a prevalence of 1% and the following genetic model: h^2^=0.85, c^2^=0, e^2^=0.15. Abbreviations: CI, confidence interval; h^2^, additive genetic effects; c^2^, shared environment effects; e^2^, unique environment effects; *r_ph_*, total phenotypic correlation; *r_ph-a_*, breakdown of the phenotypic correlation into genetic components; *r_ph-e_*, breakdown of the phenotypic correlation into environmental components; *r_g_*, genetic correlation; *r_e_*, unique environmental correlation. CIs including zero indicate non-significance. ^a^ A ∆χ2 with a significant *p* value indicates a good fit.
